# Proof of concept for the usability of the IRRAMICE for proton preclinical radiobiological research by a traceable dosimetry evaluation

**DOI:** 10.1038/s41598-025-24546-y

**Published:** 2025-11-19

**Authors:** Reem Ahmad, Richard Coos, Ileana Silvestre Patallo, Colin Baker, Christian Brunet, Nilsu Cini, Andrew Gosling, Alex Grimwood, Mohammad Hussein, Stephane Lucas, Andrew Nisbet, Hugo Palmans, Andrew Poynter, Alison Warry, Maria Hawkins

**Affiliations:** 1 Department of Medical Physics and Biomedical Engineering, University College London, London, UK; 2 Radiotherapy and Radiation Dosimetry Group, National Physical Laboratory, Teddington, UK; 3 LARN-NARILIS, University of Namur, Namur, Belgium; 4 Medical Physics Department, Proton Therapy Centre, University College Hospital, London, UK; 5 Research Unit For Analysis By Nuclear Reaction, University of Namur, Namur, Belgium; 6 Medical Physics, MedAustron Ion Therapy Center, Wiener Neustadt, Austria

**Keywords:** Dosimetry, Preclinical, Proton therapy, IRRAMICE, Radiotherapy, Radiotherapy, Biomedical engineering, Experimental particle physics

## Abstract

Radiobiology relies on animal studies to demonstrate efficacy and safety of treatments. For proton-based studies in clinical settings, bespoke solutions, enabling animal irradiations without compromising health and safety regulations, need to be developed to facilitate the research. This study aims to demonstrate the usability of the IRRAMICE, a novel system with bespoke collimators to effectively treat small targets, within proton clinical facilities. Following a clinical workflow of planning CT, treatment plan design and delivery, a dosimetric evaluation of the IRRAMICE was conducted. Traceable absorbed dose was determined by measurements with (i) ion chamber, (ii) alanine pellets within a mouse phantom and a bespoke holder in the IRRAMICE, and (iii) Gafchromic film in the holder for a relative evaluation of the collimation system. Dose determined with alanine pellets in the mouse phantom were within 2% of the planned dose. The 2%/2 mm local gamma analysis of the dose distribution of the collimated beam spot determined by the film and the treatment planning system showed an average 98.3% passing rate. Through measurements in mouse phantoms, IRRAMICE demonstrated to be a suitable device for enabling the setup and delivery of treatment plans in a reliable and reproducible manner, to facilitate in vivo preclinical experiments.

**Supplementary Information:**

The online version contains supplementary material available at 10.1038/s41598-025-24546-y.

## Introduction

Over the past five years, widespread interest in proton beam therapy (PBT) due to its highly conformal dose distribution has led to an increase in the number of clinical centres worldwide^1,2^. Small animal preclinical trials are essential to assess the efficacy and safety of proton therapy, as they represent a crucial link between preclinical (in vivo and in vitro) experiments and clinical translation. Precision is at the forefront of proton therapy advances, with developments in treatment planning and dosimetry techniques, aimed at optimizing delivery and minimizing impact on healthy tissues. Preclinical in vivo studies focus on the deterministic damage caused by protons, particularly in the spread-out Bragg peak (SOBP), discerning lesion-inducing doses but also spatial fractionation and more recently the FLASH effect.

Photon-based commercial platforms, dedicated to preclinical irradiation research, are widely accessible^3^. Currently, proton-based preclinical studies are predominantly carried out in centres with designated research rooms^4–7^, which highlights the challenges of performing PBT preclinical animal research within clinical facilities. Moreover, this underlines the importance of developing platforms for safely positioning and irradiating small animals in preclinical proton research in clinical PBT rooms, contributing to the availability of robust and reproducible in vivo data.

**Fig. 1:**
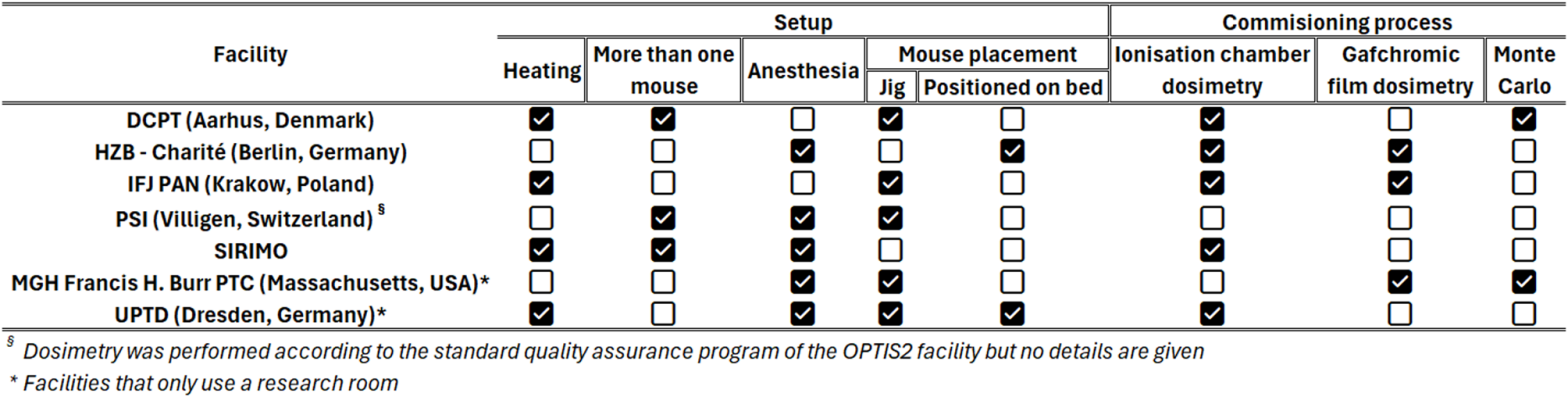
Summary of tailored approaches used by several research centres.

With no commercial options available, centres across the world have devised bespoke setups to facilitate their research. Examples are highlighted in Figure.1, summarising key aspects of their setups (details and citations in Supplementary Table 1).

Another challenging aspect of developing suitable proton experimental setups for preclinical studies, is the substantial reduction in target volumes, at least 10 times smaller, from human to mouse organ/tumour sizes. This highlights the limitations of using an uncollimated pencil beam, as the targets are both small and in close proximity to organs at risk. It should be noted that the need for smaller beam sizes, prevents dosimetry under standardised conditions and requires high positioning accuracy for beams and animals^8^. Therefore, the development of a system capable of delivering precise collimation and accurate targeting with clinically relevant high-energy proton beams is crucial for advancing future preclinical proton research. Such a device will support the harmonization of preclinical irradiation studies and help minimize variability caused by differences in spot sizes across research centres.

As part of work package 1 of a Walloon (Be) publicly funded PROTHERWAL project, the Laboratory of Analysis by Nuclear Reaction (LARN) from University of Namur (UNamur) developed a device enabling small animals to be irradiated in clinical treatment rooms. The IRRAMICE was devised as a portable device, where mice are in a sealed, contained environment, with no interactions with surfaces and air of clinical facilities or modifications to the clinical machine. A bespoke collimation system, allowing the irradiation of small targets within the animal, was also developed.

This study aims to demonstrate the capabilities of the IRRAMICE, for its use within a clinical irradiation workflow. From a dosimetric perspective, it assessed the overall effectiveness of the collimation system and differences between doses delivered to those calculated by treatment planning systems (TPS). By conducting a positional and dosimetric evaluation, with multiple dose quantification methods, the study evaluated the effectiveness of utilising the IRRAMICE in a PBT clinical room.

## Materials and methods

### IRRAMICE

**Fig. 2:**
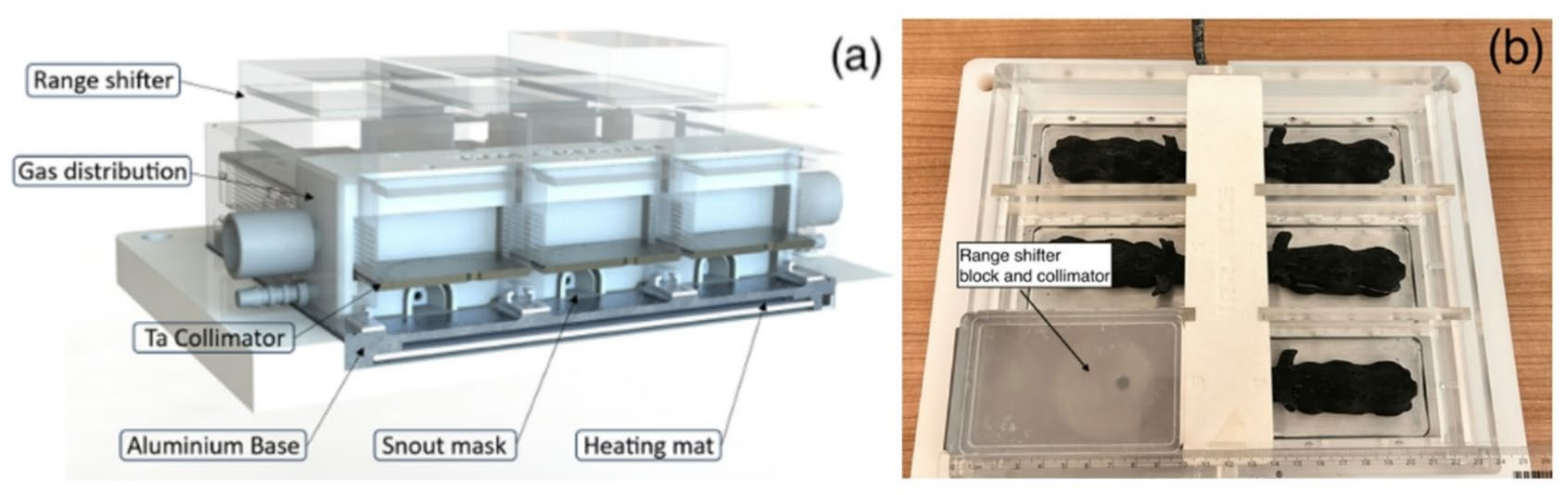
3D cross-section view of IRRAMICE (**a**) and an image of the device with six mouse phantoms, with one compartment showing the collimator and range shifter block positioned on top of the mouse compartment (**b**).

The IRRAMICE (Figure.2 and Supplementary Material) consists of a plexiglass cage attached to an aluminium base, sealed by a joint. Up to six mice can be placed in individual compartments at the bottom of the cage. The cage bottom contains an adjustable heating mat, preventing temperature changes during the procedure and mitigating risks of hypothermia. A central ramp delivers a controlled constant flow of anaesthetic gas (isoflurane) to each compartment via a mouse snout mask. Gas outlets recapture and recycle excess of isoflurane, preventing its escape from the cage, and the associated risk to the surrounding research personnel. If needed, mice kept under anaesthesia can be secured by elastic bands attached to the bottom of the cage.

The device was designed and developed to comply with relevant regulations and aspects of animal-related research: (i) animal welfare (i.e. gas anaesthesia and heated bed during treatment), (ii) the nature of the proton interaction with the device materials, including the bespoke collimation system, and (iii) portability of the device. The device was conceived for its safe use on patient treatment couches, avoiding mice direct contact and retaining all fluids potentially released. It allows the irradiation of six samples/mice per experiment.

The components enabling proton irradiation with the IRRAMICE in this study are as follows: 55 mm PMMA blocks are used as range shifters to adjust the proton energy, enabling mouse irradiation without modifying the accelerator’s clinical parameters. For targeted irradiations, individual 3 mm thick tantalum (Ta) plates are placed above each of the six compartments, acting as independent collimators. Each plate incorporates rotating discs with a small circular aperture (6 mm), which collimates the beam towards the target volume whilst shielding surrounding non-targeted regions.

The entire system is dismountable for thorough cleaning after use and can be transported on a trolley (Supplementary Figure.1).

### Mouse phantom

To model the delivery of PBT plans for the IRRAMICE dosimetric evaluation, considering situations relevant to in vivo experiments, an anatomically relevant phantom was needed. One such phantom, is the 3D printed heterogenous mouse phantom developed by Price et al.^9^. It was built using dual extrusion printing of (i) Acrylonitrile Butadiene Styrene (ABS) for the mouse body and (ii) ABS doped with CaTiO_3 _for the skeleton, with (iii) lungs as air cavities. In this study, we used the version of the phantom 3D printed by Biglin et al.^10^ which encompasses two sections, segmented across the central coronal plane (Supplementary Figure.2), and cylindrical compartments for alanine in the brain and abdomen regions.

### Irradiation workflow

#### Planning CT scan

**Fig. 3:**
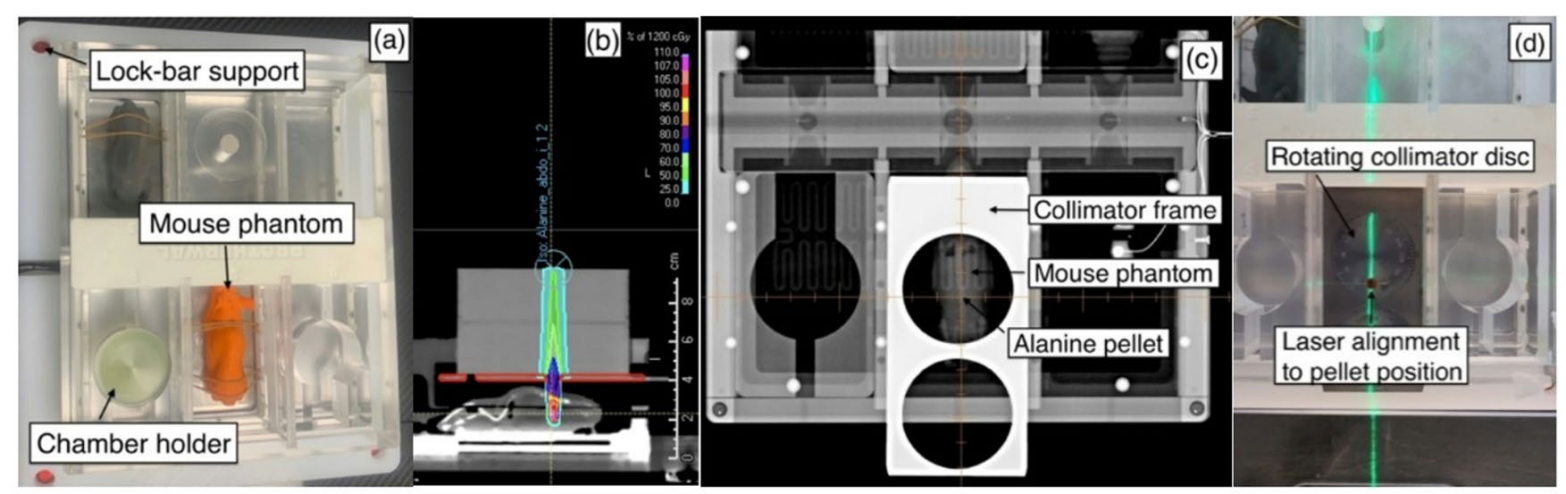
IRRAMICE setup in commissioning mode, (**a**) with phantoms and holders for ionisation chambers and alanine pellet holders, positioned using the lock-bars to ensure reproducibility. (**b**) Treatment plan with a dose of 12 Gy delivered to an alanine pellet placed in the abdomen compartment within the mouse phantom. (**c**) kV image taken without the rotating disc part of the collimator to allow for correct alignment of the pellet (lateral and longitudinal). (**d**) Lasers used to align the rotating collimator to the marker on the mouse phantom.

The clinical workflow started with a planning CT scan of the device, phantoms, collimators, and range shifter blocks in position. The IRRAMICE was scanned with a CT scanner (Philips Spectral CT 7500) (Figure.3a) at University College London Hospital (UCLH) Proton Centre. Images were imported into RayStation (v14.0, RaySearch Laboratories, Sweden), and relevant structures were segmented. During the planning CT, plastic collimators were used, as Ta caused significant imaging artefacts. These were subsequently segmented and overridden in RayStation to ensure the plan accounted for the true high density of Ta.

#### Treatment plan design

All treatment plans were calculated using Monte Carlo based RayStation algorithms (1 mm grid resolution). These plans included the range shifters, with Ta collimator placed underneath and alanine pellets placed in their respective positions (Figure.3b). These three structures were manually overridden by assigning alanine (1.000 g/cm^3^), PMMA (1.190 g/cm^3^) and Ta (16.654 g/cm^3^) mass densities, respectively. Alanine pellets were contoured as planning target volumes (PTVs). In each plan, the source to isocentre distance was set to the surface of the range shifter block, with 12 Gy prescribed to the PTV (details outlined in Supplementary Tables 2 and Supplementary Figure.3). At 10–15 Gy the uncertainty of alanine is 1.7% (k = 2) and pellet-to-pellet reproducibility is 1.0% (k = 2)^11^. The *Alanine_film_pellet_holder* plan was manually optimised to deliver a SOBP modelling an extreme case, covering a larger (in depth) PTV (Supplementary Figure.4). This plan was independently evaluated at the position of the alanine or films. The mouse phantom plans were manually optimised to homogeneously target the alanine pellet in each respective compartment.

#### Positioning verification and plan delivery

The IRRAMICE (without the rotating collimator disc) was positioned at the UCLH Proton Centre (Varian ProBeam), on the patient couch using indexed lock-bars. A cone-beam CT (CBCT) study was acquired to align the device to the treatment planning CT. The couch was subsequently shifted to ensure the position was correct in all planes. For each irradiation, it was crucial that the phantom was correctly aligned. With the couch shifted, onboard kV images were acquired, to verify the alignment of the pellet’s centre with the beam isocentre and the lasers (Figure.3c). Without altering the device position, the rotating collimator disc was then placed and aligned both visually and using lasers (Figure.3d). Finally, a second kV image was performed for a final verification of the positioning prior to delivering the plan.

### Experimental measurements

#### Reference absorbed dose measurements in solid water

Firstly, absorbed dose output in reference conditions was determined with an ionization chamber. Measurements were performed with a National Physical Laboratory (NPL) secondary standard (SS) plane-parallel ionization chamber (IC) (PTW-34001/Roos, SN:2896), connected to a calibrated PTW Unidos E electrometer (SN:92580). The chamber has a ^60^Co calibration coefficient, traceable to the UK primary standard for absorbed dose, the NPL graphite calorimeter.

The IC was placed with its effective point of measurement (EPOM) at the beam isocentre, at 2 cm depth in a 30 cm × 30 cm × 12 cm phantom of WT1 water equivalent material. The IC chamber crossline and inline alignment was checked against the positioning lasers. The phantom level and its perpendicularity to the beam were also checked. A 100 MeV single layer scanned 10 cm × 10 cm field (1681 spots, 2.5 mm spot spacing and 10 MU/spot) was used. Depth of 2 cm followed the setup used during a beam monitor calibration and reference dosimetry audit for proton beams developed by NPL and performed at UCLH in April 2021. Negative charge was collected at the recommended operational voltage of −200 V. The final uncorrected reading, $\:{M}_{Q}$ was determined as the average of four readings. Ion recombination was experimentally determined using the two-voltage method, at −100 V and − 200 V. Measurements with the ionization chamber were also corrected for variations in the environmental conditions. Temperature and pressure were measured, and the relevant correction was determined by the formalism described in the updated IAEA TRS-398^12^ (pressure 998.8 mbar, temperature 21.9 °C, with a correction of 1.021). The absorbed dose was also determined using the updated IAEA TRS-398^12^, with kQ data taken from the work by Palmans et al.^13^. The practical range (7.97 g/cm^2^), provided by UCLH, was determined from measurements of integral depth-dose curves in water and allowed for the determination of the residual range. Measured absorbed dose to water was compared to results reported by UCLH and the NPL reference dosimetry audit.

Additional measurements of absorbed dose in reference conditions with alanine detectors provided by the NPL reference dosimetry service were performed (nominal diameter and height of 5 mm and 2.3 mm respectively)^14^. Dose determination methodology and related uncertainties are presented by Sharpe et al.^11,15^. The centre of the pellet height was placed at the isocentre, at 2 cm depth in the WT1 phantom. Temperatures during the irradiations were recorded (values ranged between 22.3 and 22.9 °C). Corrections to measured electron paramagnetic resonance (EPR) signal for temperature variations were applied^16^.

A well characterized and established measurement process at NPL, allows for alanine to be considered a secondary standard detector for traceable reference dosimetry and for end-to-end dosimetry audits in high energy photon/protons beams. For this pellet size, a dose greater or equal to 10 Gy is recommended and is associated with an uncertainty of 1.7% at the 95% confidence level^17,18^. Therefore, the target dose for the alanine reference measurements was 12 Gy. Based on the IC measured reference dose, the number of Monitor Units (MU) per spot in the reference 10 cm × 10 cm field were scaled to allow for the delivery of 12 Gy to three independent alanine pellets.

Following the recommendations from the NPL Report IR 48, reported doses to alanine were corrected by the alanine cross-calibration correction factor ($\:{k}_{{Q}_{cross},{Q}_{o}}^{alanine}$) which takes into consideration its under response in proton beams compared to ^60^Co (calibration beam for the EPR signal)^18^.

The results of the reference absorbed dose measured with alanine and the NPL SS ionization chamber were compared. A good agreement between reference dose measured by alanine and the ionometric method (1%, within uncertainty of both methods) increases confidence in alanine measurements where it is not possible to utilize ionization chambers, as is the case in this study for measurements within small phantoms and for small fields.

#### Reference measurements with EBT3 gafchromic films

Pieces of 3 cm x 3 cm were cut from EBT3 film sheets (lot #01042102, Ashland). The calibration curve (CC) was previously obtained by delivering a 10 cm x 10 cm single energy layer of 170 MeV to the films placed in WT1 solid water, at 2 cm depth. The application films (AH1-AH6) and the reference films irradiated during the IRRAMICE measurement session were from the same lot as the calibration films. All films were scanned 48 h post-irradiation with an Epson Perfection V850 pro scanner. As part of the film handling procedure, the film pieces were aligned on the scanner bed, such that the scanning direction was parallel to the long edge of the film, minimizing directional artifacts and ensuring consistent optical density. A glass plate was placed on top to flatten the film and reduce any air gaps during scanning. The scanner was configured to produce a TIFF image format with no colour corrections applied. The average of 5 scanned images were processed using an in-house film analysis tool, Vigo^19^, run through MATLAB 2019b (Mathworks Inc). No additional filters were applied to the scanned images. As part of the processing, net optical density was converted to dose through a polynomial fitting using the green colour channel.

To account for differences between the beam quality used for the film calibration and our IC output measurements, the three independent reference films were irradiated with a 100 MeV 10 cm x 10 cm field. Films were marked for orientation and placed in the WT1 phantom, at the isocentre and 2 cm depth. The MU/spot in the layer of the reference field were scaled to deliver 2 Gy. The average dose at the centre of a region of interest (ROI) (10 mm x 10 mm) from each of the reference films was used to rescale the 2D dose distribution from films irradiated in the IRRAMICE (AH1-AH6).

As the EBT3 films were used to assess the alignment of the field with the target, no corrections were applied to account for LET differences. Correcting for this dependence can be complex as shown in the work by Resch et al.^20^. Nevertheless, the work of Clausen et al^8^. shows that for similar sized targets to the ones in this study, the variation of the correction as a function of depth is not large (2% over a 1 cm portion of the SOBP). Moreover, for lateral dose distributions the variation of LET is considerably lower and thus the influence of LET on the lateral profile is much smaller.

#### Alanine and EBT3 film measurements in IRRAMICE

Alanine was placed in either the PMMA holder at 2 cm depth (Supplementary Figure.5), or at two anatomical regions within the mouse phantom: (i) head and (ii) abdomen. In all cases, treatment plans were delivered in triplicate. Temperature was recorded during each irradiation. Pellets were taken to NPL for analysis.

Reported doses were initially corrected by ($\:{k}_{{Q}_{cross},{Q}_{o}}^{alanine}$)^18^. Additionally, corrections for the relative effectiveness (η) of alanine response were estimated based on the average linear energy transfer (LET) to η according to the model proposed by Herrmann^21^, describing the under-response of alanine to proton beams with multiple layers. To estimate h, the average LET over the alanine ROI for each plan was calculated using RayStation.

Films were irradiated in triplicate, inside a homogenous PMMA phantom, to assess the spot size, at two locations (Supplementary Table 2). One set was placed directly below (AH4, AH5, AH6) and the second at a physical depth of 2 cm (AH1, AH2, AH3) from the collimator (Supplementary Figure.6).

Films were stored in a light-tight envelope. A relative dose comparison was performed between the collimated beam shape, as calculated by the TPS and measured with the films. Only a relative profile comparison was conducted, as quenching corrections were not applied^22^. The TPS dose cube, exported from RayStation, included 2D dose planes at film positions, below, and at 2 cm from the collimator. In parallel, the CC was applied to each film, using Vigo to obtain the films 2D dose distributions. They were subsequently rescaled to account for differences in conditions between the CC and the application films measurements. Profiles at the centre of beam were exported. Additionally, A 2%/2 mm local gamma analysis was performed in Vigo comparing film and TPS data. From the profiles, field and penumbra sizes were compared between film and TPS data.

## Results

### Reference absorbed dose measurements in solid water

The practical proton range for 100 MeV (7.97 g/cm^2^), led to a calculated residual range of 5.97 g/cm^2^. From Table 37b of the IAEA TRS-39^12^, the PTW Roos chamber specific beam quality correction factor was 0.9964. The measured ion recombination and polarity corrections were 1.001 and 1.000 respectively (electrometer correction was unnecessary for the medium range and level of charge collected).

After applying the necessary corrections to the chamber readings, the dose measured at 2 cm depth by the 100 MeV single layer scanned 10 cm × 10 cm field was 0.583 Gy (in agreement with results from a previous dosimetry audit with a ratio of 1.000 for the two independent measurements).

The ^60^Co-reference value of absorbed dose to water reported by NPL for each alanine pellet was converted to the Q_cross_ reference value of absorbed dose to water (reference dose in the proton beam). The alanine cross-calibration correction factor ($\:{k}_{{Q}_{cross},{Q}_{o}}^{alanine}$) = 1.022 was used^18^. Average reference dose with alanine (corrected by alanine cross-calibration factor) was 12.16 ± 0.23 Gy (Combined Type A and B standard uncertainty (k = 1), considering the uncertainty of the determination of ($\:{k}_{{Q}_{cross},{Q}_{o}}^{alanine}$), as reported by Palmans et al^18^.). This was 0.997% larger than the 12.04 Gy expected by scaling the SS-IC reference output measurements. Results for individual pellets are presented in Supplementary Table 3.

### Alanine and EBT3 film measurements in IRRAMICE

The results presented here refer to the doses measured by film and alanine for the plans delivered after undergoing the clinical workflow and the alignment procedure described in the methodology section. A summary of the corrected absorbed dose to water determined with alanine in IRRAMICE and its comparison with the TPS calculated dose is shown in Table 1.

**Table 1:** Summary of the physical dose to water values from raystation TPS compared to Alanine measurements. ** corrected by Alanine cross-calibration factor. Combined (Type A and B) standard uncertainty (k = 1), considering the uncertainty of the determination of ($\:{k}_{{Q}_{cross},{Q}_{o}}^{alanine}$), as reported by Palmans et al^18^.. ^§§^ corrected by relative effectiveness. Combined (Type A and B) standard uncertainty (k = 1), considering the uncertainty of the method used for the Estimation of the corrections based on typical agreement of the response of the fully corrected Alanine results with ionization chamber measured doses in end-to-end dosimetric studies^18,23^.

Alanine position	TPS, dose (Gy)	Alanine dose ± U^**^ (Gy)	% Difference	Relative effectiveness correction (h)	Alanine dose ± U^§§^ (Gy)	% Difference
PMMA holder in IRRAMICE	10.75	10.30 ± 0.19	−4.41	0.980	10.51 ± 0.21	−2.30
Mouse (abdomen)	11.67	11.60 ± 0.26	−0.57	0.975	11.90 ± 0.28	1.94
Mouse (brain)	10.98	10.64 ± 0.22	−3.23	0.980	10.85 ± 0.24	−1.18

Applying the plan-specific relative effectiveness correction to the alanine results, leads to an average difference between the TPS-calculated and alanine-based absorbed doses of −0.51% (corrected by ($\:{k}_{{Q}_{cross},{Q}_{o}}^{alanine}$) and h). The average LET (considering the three plans) calculated with RayStation over the alanine ROI was 30.62 MeV cm^2^/g. That corresponds to an average correction to the alanine under-response of 2.14%.

**Fig. 4:**
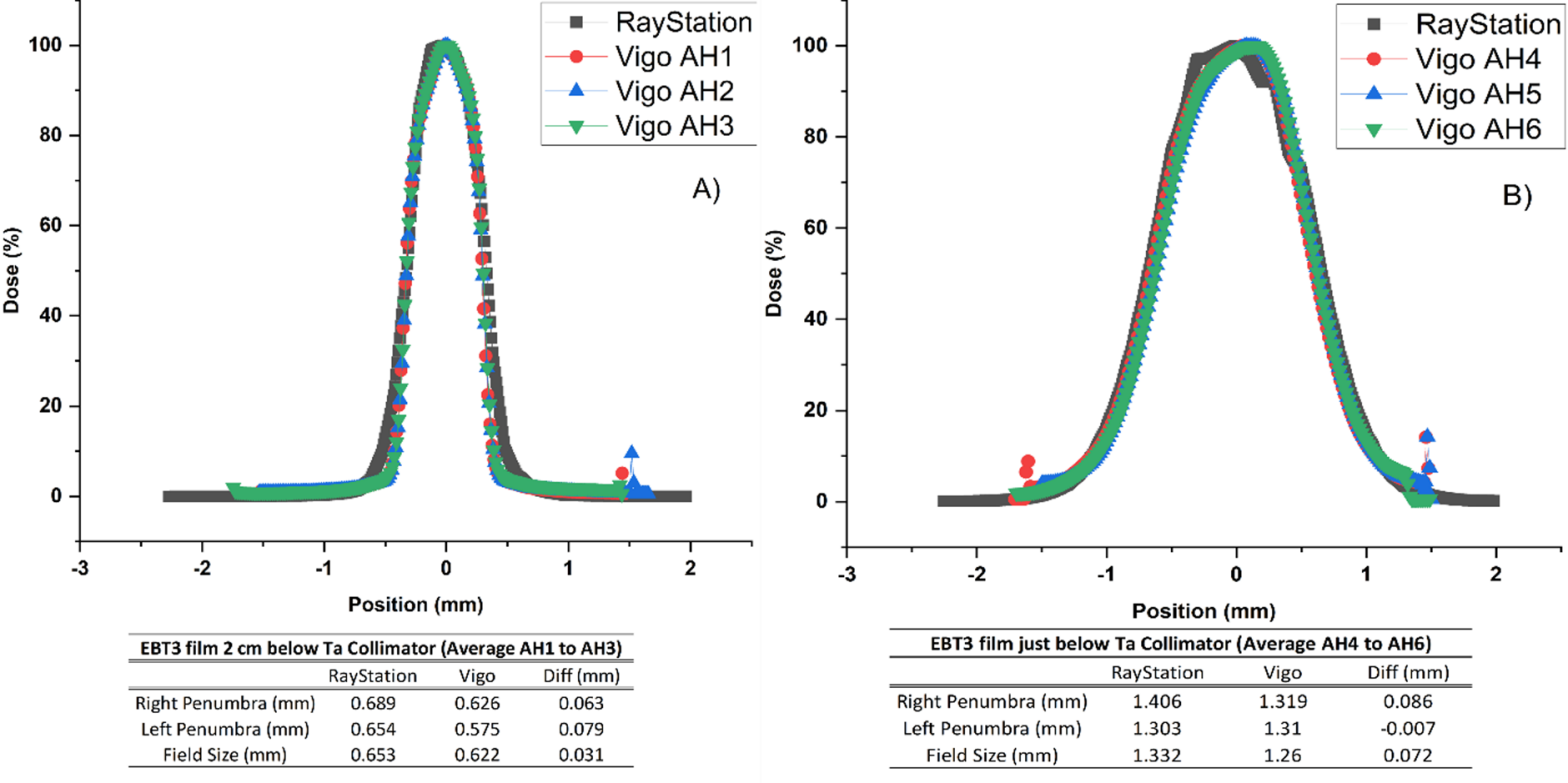
Profile comparison of TPS-calculated and EBT3 measured profiles in at (**a**) 2 cm below the Ta collimator and (**b**) immediately below the collimator within the alanine-film holder, placed in IRRAMICE.

Results comparing the TPS-calculated and measured film profiles are shown in Figure 4.

**Fig. 5:**
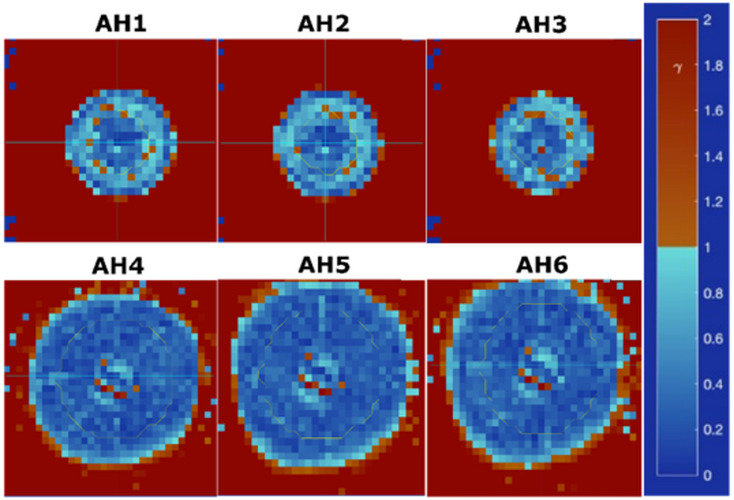
Gamma index maps for each respective film, where AH1-3 were at a 2 cm depth and AH4-6 were immediately below the collimator.

The passing rate of the local 2%/2 mm gamma analysis comparing the TPS-calculated and measured 2D dose distributions was on average 98.3%. There was no difference between the passing rates for films directly below the collimator (AH4 to AH6) and at 2 cm physical depth. An example of this analysis is shown in Figure.5.

The areas with a gamma index larger than 1 (red colour) are in the regions of the field tails. For global gamma analysis, the passing rate in the areas away from the centre of field improves, with all g ≤ 1.

## Discussion

This study presents the feasibility of using the IRRAMICE to deliver collimated proton beams to small targets, through a mouse-like phantom positioned within the device, as a real mouse would be. A comprehensive evaluation of the IRRAMICE compared the dose delivered through a clinical workflow to the dose calculated by the TPS, demonstrating the usability of the device and its capability to be used within a clinical proton facility without modifications to the clinical setup.

Measurements in the PMMA holder showed a smaller standard deviation, as it is simpler geometry (with the possibility to visualize the position of the pellet in relation to the lasers), allows for a more precise confirmation of the position of its centre in the alignment kV images, as well as the collimator aperture, in relation to the central position of the pellet.

Alanine-based reference absorbed dose measurements were within 1% of the expected value, as determined with a SS ionisation chamber. For the three analysed plans, uncorrected alanine doses were lower than calculated. Applying corrections for the relative effectiveness of the response of alanine reduced the average difference between the TPS-calculated and alanine-measured dose from − 2.74% to −0.51%. No significant differences were found for the plan-specific estimated h corrections. This was expected, considering that the plans, optimised to homogeneously irradiate the pellet volume used similar number and energy layers.

Our results are comparable to the end-to-end study for clinical radiotherapy performed by Carlino et al^23^.. They found a −1.9% difference when comparing alanine-measured and TPS-calculated dose in a homogeneous phantom and slightly larger deviations for measurements of clinically relevant plans (larger fields) in anthropomorphic phantoms. When correcting for $\:({k}_{{Q}_{cross},{Q}_{o}}^{alanine}$) and the relative effectiveness, Carlino et al. also found agreement within 2% in a dosimetric audit in six proton therapy centers^24^ As in our study, raw alanine results in both studies were lower than the TPS-calculated. Our abdomen plan showed a larger corrected alanine dose than calculated, however the results are within the uncertainty of the determination of the correction. Although Carlino’s study irradiated a larger number of pellets, like in theirs, all our dose differences were within 5%.

Films measurements confirmed the validity of the approach used to image the device with PMMA collimators and manually assigning Ta density to the structures in the TPS, avoiding imaging artefacts during the scanning process. The comparison of the TPS-calculated and film measured spot profiles, showed that for the plan delivered to the alanine-film holder, optimized to achieve a homogenous depth dose profile down up to 2.5 cm, the beam spot profile closer to the collimator was larger than at 2 cm depth. This was expected, as some energy layers exceeded the collimation energy limit. Differences between calculated and measured beam spot profile parameters (e.g. penumbra) could be attributed to the coarser resolution of the TPS dose calculation grid (1 mm) compared to the film scanning resolution (0.169 mm/pixel)^25–31^.

As shown in Figure.1, centres across the world have adopted different setups to facilitate animal irradiations, utilizing bespoke methodologies for positioning, immobilization as well as dosimetric evaluations. The use of the IRRAMICE can serve to standardize these approaches, such that reliable comparisons can be made across multiple institutions, whilst also standardizing animal welfare.

Having validated the applicability of a clinical workflow and the accuracy of the doses delivered to a mouse phantom positioned within IRRAMICE, future investigations will focus on evaluating the dose delivery workflow efficacy in an in vivo experiment with a small animal. The measurement methodology and irradiation workflow implemented here can be adapted to the verification of several collimator sizes and energy ranges. Further evaluation of alanine relative effectiveness corrections and associated uncertainties will be also performed.

## Conclusion

The IRRAMICE showed to be a suitable, versatile device to support small animal irradiations in clinical proton facilities. The device enables adequate collimation to irradiate small targets with high-energy proton beams. The system offers advantages over those which can only deliver large (uncollimated) irradiation fields. Although this work was performed in a proton centre, the simple integration with the patient couch allows for its translation to any radiation facility (e.g. x-ray linacs), providing the collimator properties are appropriately designed.

Overall, a clinical workflow was adopted and used to accurately deliver targeted doses, demonstrating the true potential of the IRRAMICE. Further work will focus on conducting and verifying the dose delivery to an animal target, in addition to evaluating multiple collimator apertures, to suit a wider range of pre-clinical small animal studies.

## Supplementary Information

Below is the link to the electronic supplementary material.

Supplementary Material 1

## Data Availability

Data is provided within the manuscript or supplementary information files.
