# Supplementary material for: Proof of concept for the usability of the IRRAMICE for proton preclinical radiobiological research by a traceable dosimetry evaluation
Source: Sci Rep. 2025 Nov 19;15:40762. doi: 10.1038/s41598-025-24546-y (PMC12630642; doi:10.1038/s41598-025-24546-y)
Supplement: Supplementary file 1 — Supplementary Material 1 [file 41598_2025_24546_MOESM1_ESM.pdf]

Supplementary materials

1. Background

Supplementary Table.1 Examples of tailored approaches used by various research centres.

Centre	Facility setup	Animal positioning/ anaesthesia	Heating	Number of Mice	Commissioning process	Target size (mm)
DCPT (Aarhus, Denmark) [7]	Research room	Unanaesthetised mice are immobalised onto a small jig with a positioning template used to ensure reproducibility.	Leg being irradiated submerged in water between 24-26°C	3-5	Absolute dosimetry with thimble and parallel plate chambers. Monte Carlo simulations used for dosimetric validation.	PTV = 10 x 3 x 3 cm^3
HZB – Charité (Berlin, Germany) [25]	Clinical room	Mice are placed directly on the patient's table and chemically anesthetized	Unknown	1	EBT3 film used to measure lateral and longitudinal properties of dose delivered. Ionization chamber used	PTV diameter = 6.3 mm
IFJ PAN (Krakow, Poland) [26]	Clinical room	Unanaesthetised mice are immobilized in a 3D printed support. Hind leg containing the tumour is usually fixed in a relatively stretched position to allow local irradiation of the tumour. As the support containing the mice is very narrow, this is sufficient to immobilize the animals during the procedure	Leg being irradiated was submerged in water at 25°C	1	Ionization chamber and slanted EBT3 film used to verify dosimetry	Right hind leg positioned in the centre of 3cm SOBP
PSI (Villigen, Switzerland) [27]	Mice positioned on a mobile metal device manipulated by a robotic chair used for patient treatment	Two coaxial tubes, strategically placed in front of the mouse's head, facilitate the administration and scavenging of anaesthetic gas	No	1	PET-Based Imaging of Tissue Activation after Proton Irradiation. Dosimetry was performed but no information was given on the detectors used. Irradiation steering files specific to each mouse were individually verified using a test phantom.	Tumor in the right shoulder with 2.5 mm extra margin.
SIRMIO project [28]	Clinical room	Inhalation anesthesia equipment and integrated heating in the mouse support bed.	Yes	1	For the beam monitoring: Segmented ionization chamber providing spatial beam profiling and total fluence	Estimated to provide spot sizes smaller than 1 mm FWHM at the

					measurement (in house developed detector)<br>For in vivo range verification: two solutions under development are ionoacoustics/US and PET imaging.	focal position at isocentre.
MGH Francis H. Burr PTC (Massachusetts, USA) [29,30]	Research room	Mice are irradiated on a specially designed holders of 4 cm width that exposes two animals simultaneously to the beam. The front legs of the mice are held in place with clothespins, while the hind legs are clamped in an appropriate system. The tail is taped to the support (this type of system is not compatible with animal welfare laws applicable in Europe). The mice were anesthetized with a mixture of Ketamine while restrained to the holder	Not mentioned	2	Monte Carlo simulations to validate the accuracy of the design of the scattering system. Dose rate was evaluated with a combination of parallel-plate ion chamber, thimble chamber, and a Faraday cup. Beam flatness was verified with EBT3 Gafchromic films. A mouse phantom and EBT3 were used for dose distribution in the mouse	Abdominal irradiation with 16 × 12 mm^2 elliptical field with a dose rate of ~120 Gy/s
UPTD (Dresden, Germany) [31]	Research room	Bespoke half-cylinder bed connected to gas anaesthesia system	Heating resistor system providing continuous flow of warm air over the mice (heating mechanism (from hot-air pulsion) at this centre differs from the one used in the device below, a choice made to limit the potential spread of germs in the treatment room.	1	Stopping power ratio of the setup (made of ABS) was measured with a Giraffe multilayer ionization chamber detector (IBA Dosimetry)	/

2. IRRAMICE

The IRRAMICE consists of a plexiglass cage attached to an aluminium base, sealed by a joint. Up to six mice can be placed in individual compartments at the bottom of the cage. The cage bottom contains an adjustable heating mat, preventing temperature changes during the procedure and mitigating risks of hypothermia. A central ramp delivers a controlled constant flow of anaesthetic gas (isoflurane) to each compartment via a mouse snout mask. Gas outlets recapture and recycle excess of isoflurane, preventing its escape from the cage, and the associated risk to the surrounding research personnel. If needed, mice kept under anaesthesia can be secured by elastic bands attached to the bottom of the cage.

This device is placed on a trolley (Supplementary Figure.1) with additional elements. The trolley's lower level holds a commercial isoflurane injection system coupled with a medical-grade oxygen bottle for gas diffusion to the ramp, in the central part of the device. The bottle is connected to oxygen flow regulators at the isoflurane pump's entrance. Excess oxygen-isoflurane mixture is recaptured from the ramp to a scavenging unit, allowing isoflurane capture. In the event of an unexpected awakening of a mouse, an anaesthesia induction chamber is present to re-anesthetize on-site. It is important to note that this system serves as a backup, as mice will be anesthetized in the animal facility, not in the treatment room. All components are arranged on the trolley and transported from the animal experimentation room to the treatment room. This mobility necessitates energy autonomy, fulfilled by a UPS battery capable of supplying 230V and ensuring the device operation during transport. Thus, the only manipulation performed in the treatment room will be the transfer of the device (containing anesthetised mice) from the trolley to the patient couch and vice versa.

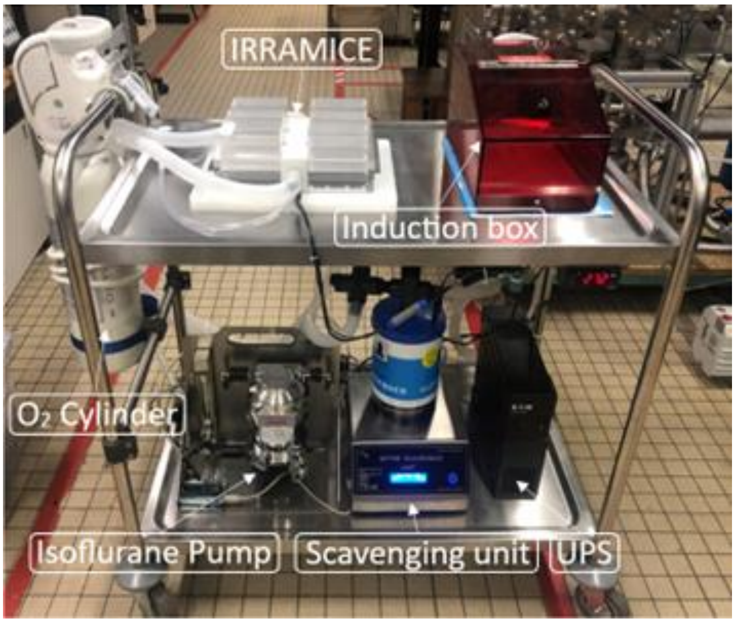

Supplementary Figure.1 Trolley with the IRRAMICE and additional components

With regards to the device itself, the IRRAMICE was tested with a specific collimator aperture size and a range shifter block thickness suited to this study. These can be adapted to suit different investigations, where future studies will include a wider range of apertures and range shifters thicknesses aiming to encompass targeting different regions of the mouse, as required by preclinical researchers.

3. Mouse phantom

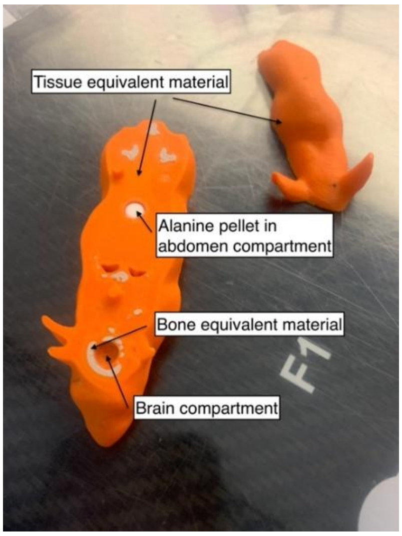

Supplementary Figure.2 Radiologically relevant mouse phantom with pellet placed in the abdomen compartment.

4. Treatment plans, beam characteristics and schematics

Supplementary Table.2 Parameters used for each respective treatment plan.

Target	Plan	Energy Layers	Energy (Mev)	Range
EBT3_Film_bellow-Ta-collimator@0cm-depth	Alanine_film_pellet_holder	8	90-116	
EBT3_Film@2cm-depth				
Alanine_Pellet@at 2cm-depth	Alanine_film_pellet_holder	8	90-116	
Pellet-in-brain	Alanine_brain_iso_moved	3	96-103	
Pellet-in-abdomen	Alanine_abdo_iso_moved	3	96-103	

Supplementary Figure.3 shows measured pristine Bragg Peak Curves and Spot Scans for the energy levels in the range that were used for the irradiation of film and alanine in the PPMA holder and the mouse phantom.

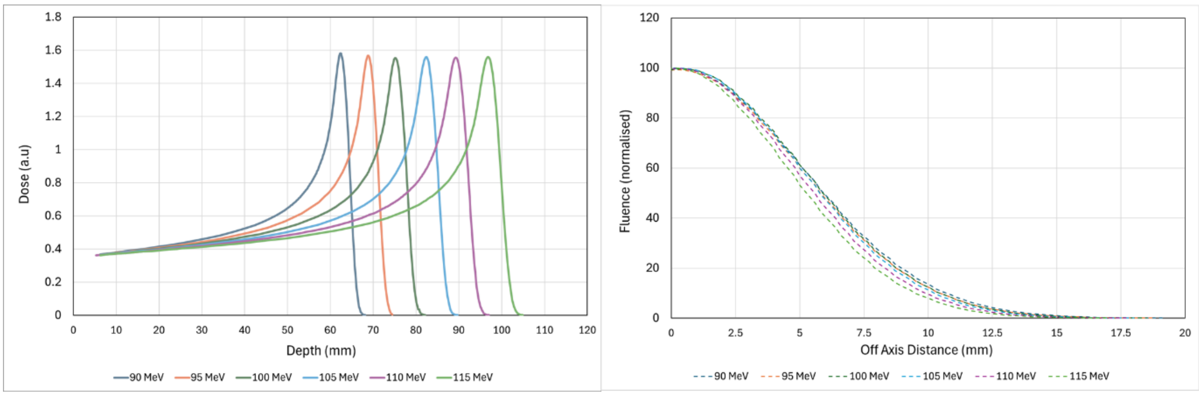

Supplementary Figure.3 Characteristics of the proton beams, covering the range of energies used for this work. Left: Pristine Bragg Peak Curves measured in a water phantom. Right: in-air half scan of the beam spots at the position of the isocentre (cross-line direction).

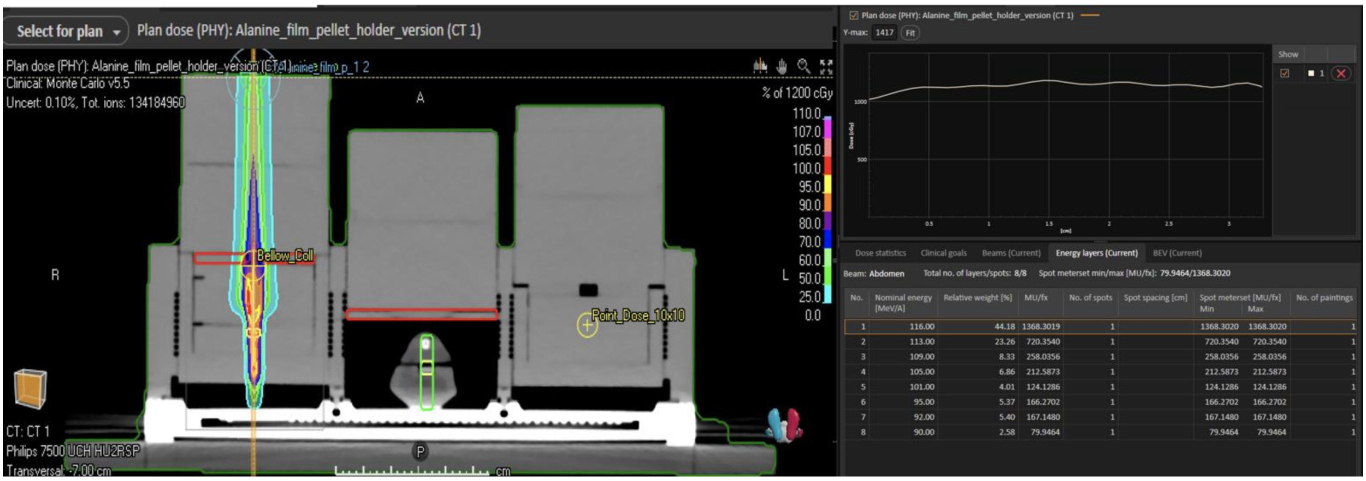

Supplementary Figure.4 Treatment plan demonstrating the homogeneity of the dose delivered to PMMA holder (top-right), optimised using 8 energy layers (bottom-right) to cover the entire depth of the holder (left), with an alanine pellet placed at a 2 cm depth to measure the dose.

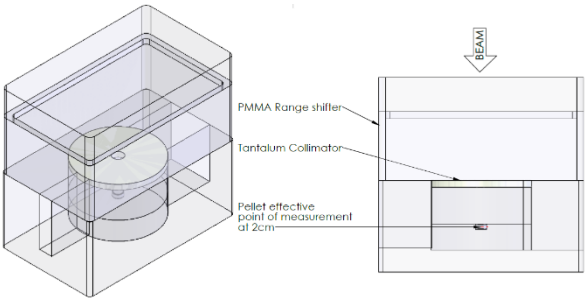

Supplementary Figure.5 Alanine placed within a PMMA holder at a 2 cm depth.

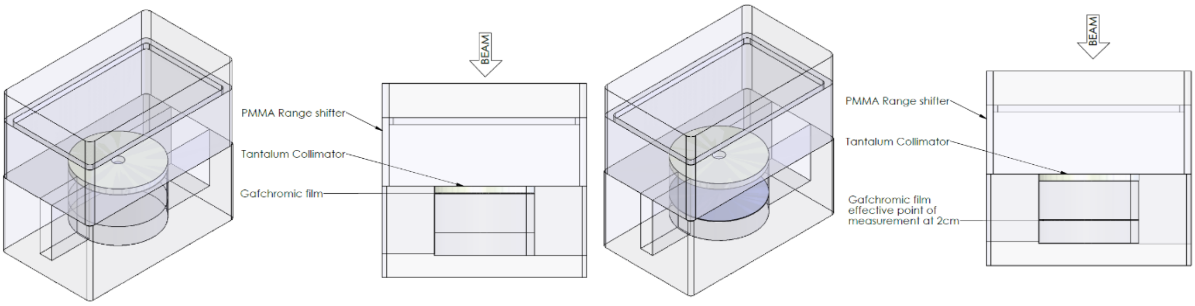

Supplementary Figure.6 Film placement immediately below the collimator and at a 2cm depth

Supplementary Table.3 Results of the reference absorbed dose measured with alanine with the calculated percentage difference (% difference was determined as: 1-(calculated dose)⁄(measured dose)).

Pellet number	Calculated dose (Gy)	Measured dose (Gy)	Difference (%)
78/1560	12.04	12.09	0.39
78/1561	12.04	12.18	1.13
78/1562	12.04	12.21	1.37
